# Bioprinting Soft 3D Models of Hematopoiesis using Natural Silk Fibroin‐Based Bioink Efficiently Supports Platelet Differentiation

**DOI:** 10.1002/advs.202308276

**Published:** 2024-03-21

**Authors:** Christian Andrea Di Buduo, Marco Lunghi, Volodymyr Kuzmenko, Pierre‐Alexandre Laurent, Giulia Della Rosa, Claudia Del Fante, Damian Edward Dalle Nogare, Florian Jug, Cesare Perotti, Koji Eto, Alessandro Pecci, Itedale Namro Redwan, Alessandra Balduini

**Affiliations:** ^1^ Department of Molecular Medicine University of Pavia Pavia 27100 Italy; ^2^ CELLINK Bioprinting AB Gothenburg 41276 Sweden; ^3^ Immunohaematology and Transfusion Service I.R.C.C.S. Policlinico S. Matteo Foundation Pavia 27100 Italy; ^4^ Human Technopole Milan 20157 Italy; ^5^ Department of Clinical Application Center for iPS Cell Research and Application (CiRA) Kyoto University Kyoto 606‐8507 Japan; ^6^ Department of Regenerative Medicine Graduate School of Medicine Chiba University Chiba 260‐8670 Japan; ^7^ Department of Internal Medicine I.R.C.C.S. Policlinico S. Matteo Foundation and University of Pavia Pavia 27100 Italy; ^8^ Department of Biomedical Engineering Tufts University Medford MA 02155 USA

**Keywords:** bioprinting, bone marrow, eltrombopag, hematopoiesis, megakaryocyte, platelet, silk

## Abstract

Hematopoietic stem and progenitor cells (HSPCs) continuously generate platelets throughout one's life. Inherited Platelet Disorders affect ≈ 3 million individuals worldwide and are characterized by defects in platelet formation or function. A critical challenge in the identification of these diseases lies in the absence of models that facilitate the study of hematopoiesis ex vivo. Here, a silk fibroin‐based bioink is developed and designed for 3D bioprinting. This bioink replicates a soft and biomimetic environment, enabling the controlled differentiation of HSPCs into platelets. The formulation consisting of silk fibroin, gelatin, and alginate is fine‐tuned to obtain a viscoelastic, shear‐thinning, thixotropic bioink with the remarkable ability to rapidly recover after bioprinting and provide structural integrity and mechanical stability over long‐term culture. Optical transparency allowed for high‐resolution imaging of platelet generation, while the incorporation of enzymatic sensors allowed quantitative analysis of glycolytic metabolism during differentiation that is represented through measurable color changes. Bioprinting patient samples revealed a decrease in metabolic activity and platelet production in Inherited Platelet Disorders. These discoveries are instrumental in establishing reference ranges for classification and automating the assessment of treatment responses. This model has far‐reaching implications for application in the research of blood‐related diseases, prioritizing drug development strategies, and tailoring personalized therapies.

## Graphical abstract

The research focuses on developing a bioink specifically tailored to replicate the softness of the hematopoietic niche within the bone marrow microenvironment to create a simplified and standardized model for in‐depth investigation of hematological disorders. Printing human hematopoietic progenitors in the silk bioink has extended their lifespan and promoted their full functionality, enabling their differentiation into megakaryocytes, and significantly improving platelet generation ex vivo.

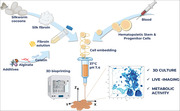

## 1 Introduction

The maturation of all blood cells occurs within the complex and dynamic bone marrow microenvironment, a soft tissue that fills the cavities of the bones, composed of a network of extracellular matrix (ECM) components and intercellular fluids generated by pressure gradients from the vasculature, facilitating the process of hematopoiesis.^[^ ^1^ ^]^ The bone marrow stiffness is highly spatially divergent, presenting Young's modulus ranging from 0.1 to 10 kPa.^[^ ^2^ ^]^ The main contributors to local viscoelasticity are proteoglycans, glycosaminoglycans, and fibrous and matricellular proteins.

Despite its non‐uniform shape, bone marrow possesses defined biophysical properties that play a crucial role in regulating the function of hematopoietic stem and progenitor cells (HSPCs). Through interactions with integrins and mechanosensitive receptors, HSPCs can sense and respond to both mechanical forces and biochemical signals present in the extracellular environment.^[^ ^3^, ^4^ ^]^ Ultimately, these regulatory mechanisms influence critical processes such as lineage commitment and maturation.

Significant progress has been made in hematopoietic stem cell research, enabling the in vitro replication of the differentiation process into blood cells. Moving from 2D cultures toward relevant tissue‐engineering approaches, 3D functional mimics of specific features of the bone marrow niche have been developed to enable mechanistic studies of physiological hematopoiesis and allow the functional interrogation of pathological states, using a variety of biomaterials, including synthetic and natural biopolymers, such as methylcellulose and hyaluronic acid.^[^ ^5^ ^]^

In recent years, the need to standardize the fabrication process and produce simple 3D culture systems advanced in parallel with the development of additive manufacturing technologies. 3D bioprinting represents a cutting‐edge technique for fabricating tissue models that closely mimic the essential properties of their in vivo counterparts.^[^ ^6^ ^]^ Bioprinting enables the controlled design of 3D constructs integrating living cells, bioactive molecules, and cytokines within biomaterials‐based bioink as scaffolds.^[^ ^7^ ^]^ Identifying bio‐printable formulations having tailored mechanical, physical, and chemical cues, that mimic functional features of the tissue of interest, is challenging. Hydrogel‐based bioink, composed of biocompatible polymers, are commonly employed due to their favorable rheological properties that support cell viability and function.^[^ ^8^ ^]^ Though, synthetic material‐based hydrogels typically exhibit low cell adhesion capacity, while natural material‐based hydrogels often lack mechanical stability, posing challenges in achieving desired 3D matrix structures during long‐term cultures. Commercially available bioink formulations do not match the extremely low stiffness of the native bone marrow niche to support hematopoiesis ex vivo. Limited 3D bioprinting approaches for HSPCs have been tempted.^[^ ^9^ ^]^ Using these models, programmable HSPC differentiation has never been achieved.

Silk fibroin, a natural protein derived from *Bombyx mori* cocoons, is used in biomedical applications, including inkjet printing and 3D bioprinting.^[^ ^10^ ^]^ Advantageous features of silk fibroin include aqueous and ambient processing conditions, robust mechanical features, and its ability to well‐stabilize labile molecules while maintaining long‐term functionality.^[^ ^11^, ^12^ ^]^ Importantly, silk biomaterials have been approved by the Food and Drug Administration for use in medical products. Previous studies by our group have demonstrated the use of custom‐made silk fibroin‐based scaffolds to allow human platelet production ex vivo.^[^ ^13^, ^14^, ^15^, ^16^ ^]^ In this study, we present a pioneering advancement in the field by developing a reproducible and physiologically relevant 3D‐bioprinted model of hematopoiesis.

One major challenge in the reconstruction of soft tissues using 3D bioprinting is to develop a material system with both optimal rheological properties for the extrusion process and ideal structural stability for its long‐term maintenance in 3D culture. To meet this need we designed a silk‐based bioink formulation including gelatin as a bulking agent and sodium alginate as an additive to induce physical crosslinking. The formulation was fine‐tuned to obtain a viscoelastic, shear‐thinning, thixotropic bioink with a remarkable ability to rapidly recover upon the deformation induced by the 3D bioprinting extrusion process. Our approach to 3D bioprinting utilizing the novel silk bioink facilitated the fabrication of multiple 3D constructs with programmable sizes, heights, and shapes, within a short timeframe and with meticulous control over temperature and pressure throughout the process. We demonstrated that the 3D‐bioprinted construct was stable under physiological culture conditions and matched the mechanical properties of the native bone marrow tissue. Silk fibroin played a crucial role in maintaining the bioink's shape, softness, and transparency, influencing 3D construct physico‐mechanical properties and preserving the bioactivity of enzymes incorporated into the formulation. These features, together with proper control of cytokine and nutrient composition, allowed the establishment of an optimal environment for preserving cell viability and differentiation into platelets. The valuable performance of the silk bioink as a 3D model of physiological hematopoiesis fostered its application toward a pre‐clinical approach for studying diseases and drug testing. Specifically, by incorporating patient samples, we leveraged the silk bioink to develop a simplified, highly reproducible, personalized platform for studying platelet disorders within a controlled laboratory environment. This integrated system allowed the simultaneous assessment of various diagnostic parameters, such as megakaryocyte metabolism, platelet production and morphology, and responses to drugs.

## 2 Results and Discussion

### 2.1 Designation and Characterization of the Silk‐Based Bioink Formulation

**Figure 1:**
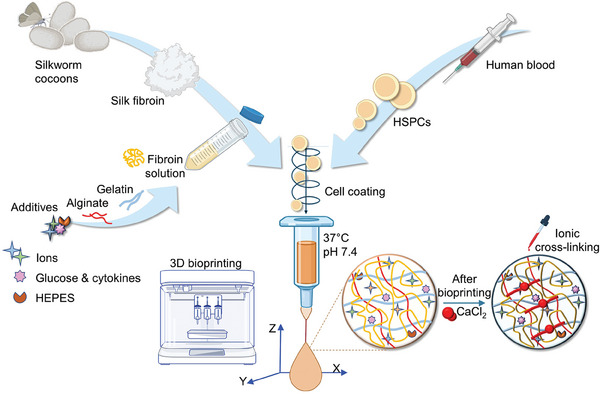
Workflow of silk bioink production and usage. Schematic representation of silk bioink preparation. Regenerated silk fibroin from natural *Bombyx mori* silkworm cocoons is first solubilized and then blended with gelatin and alginate. Electrolyte balance is guaranteed by the addition of ions. Glucose and cytokine feeding is used to provide fuel to cell metabolism and differentiation, both during and after the bioprinting process. Cells are mixed directly inside the bioink before the bioprinting process that takes place at 37 °C using a heated printhead. After the bioprinting, ionic cross‐linking stabilizes the 3D structure‐supporting pattern. Made using BioRender.com.

HSPCs are fragile and require a defined chemico‐physical and mechanical environment to survive and differentiate outside the human body.^[^ ^3^ ^]^ A tailored bioink composition was designed to support the bioprinting of blood progenitor cells at physiologically relevant conditions (37 °C, pH 7.4) and provide a stable biomimetic 3D microenvironment sustaining their survival, differentiation, and maturation. Specifically, a solution of regenerated silk fibroin, from natural *Bombyx mori* silkworm cocoons, was blended with gelatin, a common biopolymer that undergoes thermo‐reversible sol‐gel transition, and sodium alginate, an algae‐derive polysaccharide able to form a gel by ionic crosslinking. Glucose feeding was used to provide fuel to cell metabolism; HEPES served as a buffering agent. This formulation will hereafter be referred to as the “silk bioink” (**Figure** **1**).

**Figure 2:**
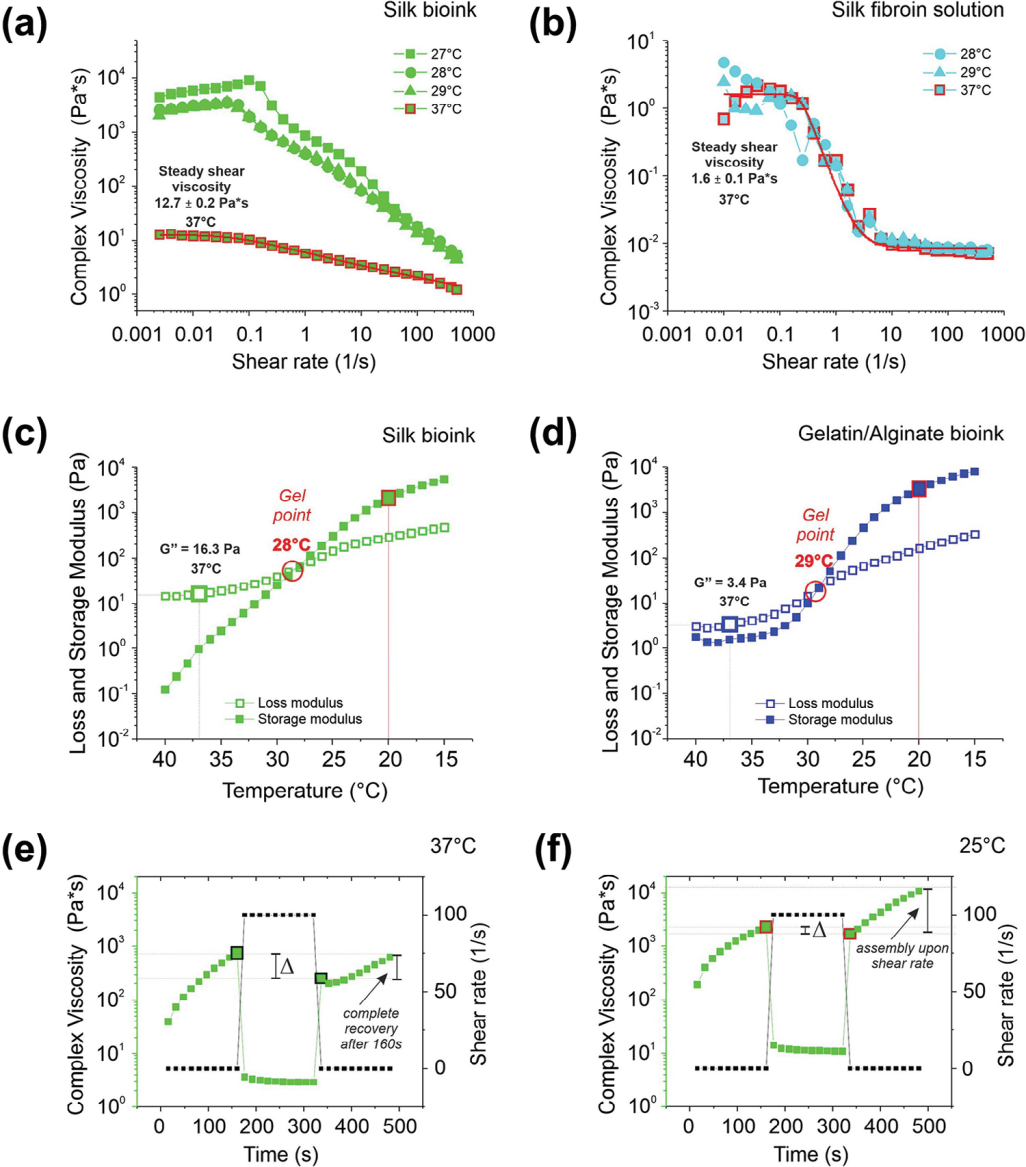
Rheological characterization. a,b), Flow sweep was performed at different temperatures to evaluate the complex viscosity as a function of the shear rate of a), silk bioink, and b), silk fibroin solution. Red lines are the values of complex viscosity at 37 °C fitted with the Carreau–Yasuda model. Representative of three independent experiments. c,d), Temperature ramp tests showed the behavior of loss (G’’) and storage (G’) modulus as a function of temperature decrease from 40 to 15 °C of (c), silk bioink and (d), gelatin/alginate bioink. The crossover point (G’ = G’’) indicates the gel point (red circle). e,f), Thixotropic test was performed to monitor silk bioink complex viscosity during a deformation and a recovery phase at (e), 37 °C and (f), 25  C. The analysis was performed by applying an initial shear rate of 0.01 1 s^−1^ for 160 s, which was ramped up to 100 1 s^−1^ for 160 s (deformation phase) and finally ramped down to 0.01 1 s^−1^ (recovery phase). The Δ indicates the recovery step after the deformation process. Representative of three independent experiments.

The rheological properties of the silk bioink were assessed by rotational rheology and compared to a silk fibroin solution and/or a bioink formulation containing only gelatin and sodium alginate, hereafter referred as the “gelatin/alginate bioink”. The shear thinning behavior was first investigated by flow ramp. The complex viscosity was monitored at different temperatures as a function of shear rate in the range of 0.001– 500 1 s^−1^. The steady shear viscosity of the silk bioink (**Figure** **2**a) was higher than the silk fibroin solution (Figure 2b), because of the mechanical contribution of gelatin and alginate to the formulation. Temperature‐dependent complex viscosity was observed only for the silk bioink due to the gelling properties of gelatin (Figure 2a,b). However, at physiologically relevant conditions (37 °C) the values of complex viscosity of silk bioink suggested a bioprinting‐suitable shear thinning behavior (steady shear viscosity = 12.74 ± 0.19 Pa^*^s, infinite viscosity = 0.43 ± 0.42 Pa^*^s and viscosity at shear rate of 100 1 s^−1^ = 2.06 Pa^*^s). The temperature sweep test (from 40 to 15 °C) was performed to determine bioink gelling temperature, which is the temperature at the crossover point (*G’ = G’’*). The silk bioink had a distinct gel point ≈ 28 °C (Figure 2c), a value slightly slower than the gelatin/alginate bioink (Figure 2d), thus suggesting a weak contribution of silk fibroin in gelatin gelling properties. At physiologically relevant temperature (37 °C), the silk bioink showed a higher viscous component (G’’ ≈ 16 Pa) with respect to gelatin/alginate alone (G’’ ≈ 3 Pa), due to the contribution of silk fibroin (Figure 2c,d). To evaluate the thixotropic behavior of the silk bioink, its complex viscosity was measured at 37 and 25 °C as a function of a sharply switched shear rate from 0.01 1 to 100 1 s^−1^ (Figure 2e,f). Silk bioink showed a typical thixotropic behavior at both temperatures; however, at 37 °C silk bioink completely recovered after 160 s (see the Δ range in Figure 2e), whereas at 25 °C upon the deformation step the values of complex viscosity increased (see the Δ range in Figure 2f). Overall, these data demonstrated that the silk bioink at 37 °C is a viscoelastic, shear‐thinning, thixotropic fluid with a remarkable ability to completely recover its structural and mechanical properties upon a deformation, ideal features for its application in extrusion‐based bioprinting.

### 2.2 Defining the 3D Bioprinting Strategy for the Silk Bioink

**Figure 3:**
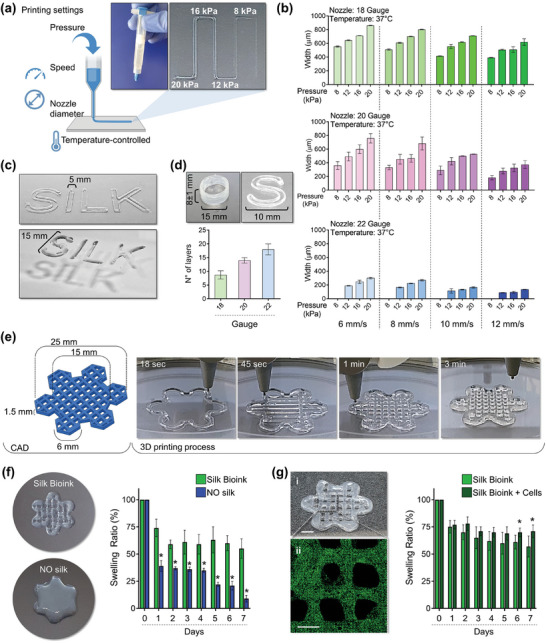
Silk bioink printability. a), Printing pressure, speed, nozzle diameter, and temperature determine the resolution of a filament extrusion‐based 3D printer. Cartoon was made using BioRender.com b), The silk bioink can be 3D printed using needles of different diameters 18G (top, green), 20G (middle, pink), and 22G (bottom, blue), at various printing speeds (6,8,10,12 mm ^−1^s) and pressures (8,12,16,20 kPa) (n = 3). c), The silk bioink can be extruded to obtain constructs of tailored widths and heights. d), 3D printing with a 20 G nozzle at 8–10 mm ^−1^ s speed, with 12–16 kPa pneumatic pressure, ensure a good balance of print fidelity and accurate deposition to obtain 3D constructs having a height with stable junctions between the layers (n = 3). e), Model (left) of a layered grid hexagon intersecting smaller hexagons at its borders designed to model a “*flower*”‐like shape. The 3D printing process into a petri dish is shown (right). f), The presence of silk in the bioink formulation is crucial for ensuring printing fidelity when compared to the same formulation without silk (left). The analysis of silk bioink swelling ratio compared to bioink formulations without silk (NO silk) is shown (right) (n = 3, ^*^ *p*<0.01). g), Confocal microscopy analysis of HSPCs after bioprinting into the silk bioink (i: scale bar = 4 mm; ii: green = CD34; scale bar = 2 mm) (left). The analysis of silk bioink swelling ratio in the presence or absence of HSPCs is shown (n = 3, ^*^ *p*<0.05).

The standardization of procedures is imperative when developing new ecosystems for preclinical studies and precision medicine.^[^ ^6^, ^17^ ^]^ Conventional manufacturing techniques, such as manual scaffolding and pipetting, fall short in achieving comparable thermostability, controllable motion, and precise deposition. Our primary reason for developing the silk bioink was to enable standardized protocols for 3D bioprinting of soft compositions, whose stability over long‐term culture is generally considered challenging.^[^ ^18^ ^]^ Rheological analysis indicated that the silk bioink should be compatible with a smooth 3D bioprinting process through extrusion using a heated nozzle at 37 °C and deposition onto a cool print bed (<25 °C) to initiate thermosetting. To prove this, a systematic set of filament extrusion experiments was performed (**Figure** **3**a). Silk bioink could be printed into sterile Petri dishes or classic multi‐well culture plates using needles of different diameters at programmable printing speeds and pneumatic pressures (Figure 3b), crucial parameters to achieve the automated production of 3D constructs of controllable widths and heights. Printing with a 20 G nozzle at 8–10 mm ^−1^ s speed, with only 12–16 kPa pneumatic pressure, was the optimal condition for obtaining a good balance of printing fidelity and accurate deposition of 3D constructs having variable shapes and heights with stable junctions between the layers (Figure 3c,d).

We next explored whether this strategy would enable the effective deposition of complementary layers of silk bioink to form 3D constructs of complex geometries encompassing challenging features such as overhangs and overlapping networks. The bone marrow niche is characterized by a highly porous nature that accommodates essential blood vessels for nutrient and oxygen distribution. We opted to fabricate a 3D model featuring an internal grid pattern mimicking this microvascular network to facilitate the exchange of nutrients, oxygen, and waste products within the printed tissue, thereby enhancing cell survival and function. This methodology enabled the creation of a layered hexagonal grid intersecting smaller hexagons at its borders to simulate a “*flower*”‐like structure (**see** Figure 3e; Video S1, Supporting Information). Multiple models could be printed at the same time (Video S2, Supporting Information). We successfully 3D bioprinted “*flowers*” customized to encompass a wide range of sizes (refer to Figure S1, Supporting Information). The shape of the printed constructs was highly consistent and matched the input values with excellent extrusion consistency and structural stability throughout the printing process (Figure 3e). This result was in stark contrast to the bioink without silk which rapidly coalesced upon deposition resulting in the fusion of layers to form structures of poorly defined features (Figure 3f). The deposition of 3D layers failed in the absence of gelatin (data not shown). The gelled 3D constructs were soon after immersed in a saline buffer solution containing CaCl_2_ to cross‐link alginate. Ionic crosslinking stabilizes the structure of 3D printed constructs which remain intact even after repeated stretching (Video S3, Supporting Information). The analysis of the swelling ratio of the 3D printed constructs, when incubated in culture conditions, demonstrated slow mass transfer, with the structure remaining within 60% of its preincubation dimensions after 7 days (Figure 3f). A significantly decreased swelling ratio was observed for the formulation without silk fibroin, likely due to the liquification of gelatin during the incubation (Figure 3f). For constructs lacking alginate, rapid dissolution into the culture medium occurred (data not shown). These results demonstrated that thermo‐responsive gelatin can be considered the sacrificial material necessary to provide excellent structural stability upon deposition onto the print bed. Right after, the swift ionic crosslinking of alginate is needed to stabilize the desired 3D structure‐supporting pattern. Overall, silk fibroin is the core material that ensures appropriate viscoelasticity during extrusion, shape fidelity during deposition, and the maintenance of a stable 3D shape/microstructure during long‐term culture.

Confocal microscopy reconstruction showed that cells were homogeneously distributed into the 3D structure (Figure 3g), and the integrity of the silk bioink was maintained during the culture, with no major changes of the swelling ratio (Figure 3g). The “*flower*”‐like design allowed selective separation of the petals to perform intermediate analyses (Figure S2a, Supporting Information). The live/dead staining, performed immediately after HSPC bioprinting, revealed a highly viable population (Figure S2a, Supporting Information). Most importantly, we developed a bespoke buffer for dissolving the 3D construct (Figure S2b, Supporting Information). When HSPCs were retrieved from the dissolved construct, cell viability was well preserved, with no significant differences over time (e.g., day 2 (93 ± 3%) and day 7 (93 ± 4%)) (Figure S2c, Supporting Information).

### 2.3 3D Bioprinting and Differentiation of Human HSPCs into Megakaryocytes

Advanced cell culture techniques and biomaterials have led to the development of versatile 3D models of hematopoiesis to support HSPC function ex vivo.^[^ ^19^ ^]^ However, engineered constructs based on porous scaffolds may show drawbacks such as a lack of control over stiffness, shape, and cell distribution, with consequent impact on cell behavior.

**Figure 4:**
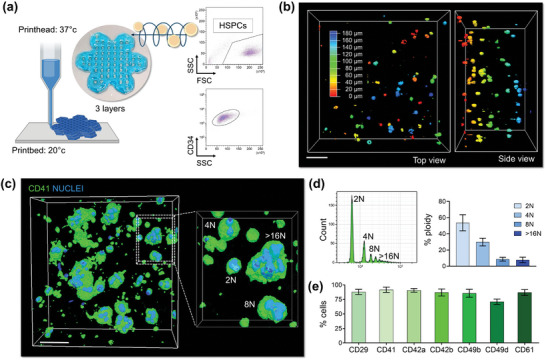
HSPC bioprinting and differentiation into the 3D silk‐based construct. a), Primary human blood progenitor cells are mixed with the silk bioink and 3D bioprinted into a 3‐layer “*flower*” construct. The flow cytometry analysis of CD34^+^ cells before 3D bioprinting is shown. Cartoon was made using BioRender.com b), Confocal microscopy analysis of cells after bioprinting (pseudo colors are used to indicate cell distribution; scale bar = 60 µm). c), 3D confocal reconstruction of polyploid megakaryocytes cultured into the silk bioink (green = CD41; blue = nuclei; scale bar = 40 µm, representative of three independent experiments). d), Flow cytometry analysis of the ploidy of megakaryocyte retrieved from the silk bioink using the dissolution buffer (n = 3). e), Percentage of megakaryocytes expressing lineage‐surface markers as assessed by flow cytometry analysis of samples retrieved from the 3D construct (n = 3).

Silk has been long proven to be a hemocompatible and low‐thrombogenic biomaterial.^[^ ^13^, ^20^ ^]^ Our group has demonstrated that silk fibroin from *Bombyx mori* silkworm cocoons represents a useful biomaterial for engineering physiologically relevant microenvironments supporting the differentiation of human megakaryocytes, the platelet progenitors.^[^ ^13^, ^15^ ^]^ Silk possesses unique properties developed through evolution to protect silk‐producing organisms.^[^ ^21^ ^]^ By embedding human HSPCs within the silk bioink, we have translated these properties to support their viability and function. Primary human CD34^+^ progenitor cells were evenly coated by the silk bioink and printed into the “*flower*”‐like construct as described above (**Figure** **4**a). The 3D reconstruction of the culture showed that cells were engrafted throughout the thickness and length of the silk bioink (Figure 4b; Video S4, Supporting Information). While 3D imaging demonstrated a living cellular microenvironment where cells, when cultured in the presence of Thrombopoietin (TPO), transformed from diploid progenitors to polyploid megakaryocytes, phenotypically analogous to those observed in the native bone marrow tissue and expressing lineage‐specific differentiation markers (Figure 4c,d). An in‐depth flow cytometry analysis of the cells, retrieved from the silk bioink, displayed the expression of the full spectrum of megakaryocytic surface markers, including >85% cells expressing CD29, CD41, CD42a, CD42b, CD49b, CD49d, and CD61 (Figure 4e). The transcript of genes for extracellular matrix components (e.g., fibronectin, type III collagen, and type IV collagen), and matrix remodeling enzymes (e.g., MMP2 and MM9), was increased over the course of maturation (Figure S3a, Supporting Information), demonstrating the physiological cell adaptation to the niche mimic which faithfully replicates the process of megakaryocyte differentiation as it happens within the human body.^[^ ^22^ ^]^

### 2.4 Spatiotemporal Volumetric Imaging of the Living Silk Bioink Construct

**Figure 5:**
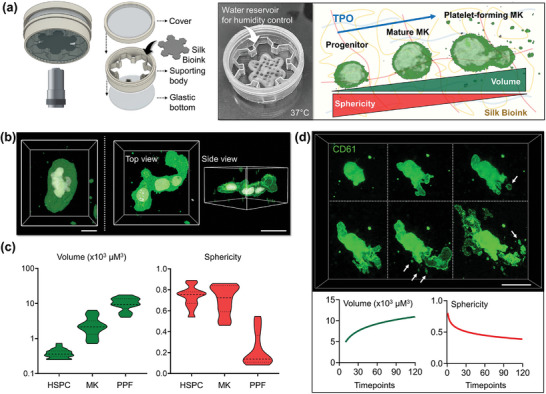
3D bioprinted silk bioink supports functional platelet production. a), A custom‐made “*flower*‐*holder*” was used for performing live‐image analysis (MK = megakaryocyte). b), 3D confocal reconstruction of polyploid megakaryocytes differentiated into the silk bioink (green = CD41; white = nuclei; scale bar = 20 µm) and c), Evaluation of single cell volume and sphericity during differentiation (HSPC = hematopoietic stem and progenitor cell, MK = megakaryocyte; PPF = proplatelet‐forming megakaryocyte, n = 100). d), Spatiotemporal volumetric imaging of samples in the last phase of differentiation shows the process of proplatelet formation. Image segmentation analysis demonstrates that megakaryocytes increase their volume while decreasing sphericity during proplatelet formation. Arrows indicate platelets released from branching filaments (green = CD61; scale bar = 30 µm).

The interconversion of the megakaryocyte cytoplasm into platelets is the most dynamic and distinctive feature of the whole hematopoiesis. Current knowledge on active hematopoiesis, including the detachment of platelets from megakaryocytes, can be only inferred by the intravital imaging of mouse bone marrow.^[^ ^23^ ^]^ Having demonstrated that silk bioink is capable of programming megakaryocyte differentiation, we next investigated whether it could instruct spatiotemporal control over platelet formation. We fabricated a live‐imaging chamber for direct visualization of the 3D culture at the microscopy stage. A water reservoir was used to keep the humidified atmosphere over long‐term visualization (**Figure** **5**a). High‐resolution imaging at the single‐cell level demonstrated that, during maturation, megakaryocytes undergo a substantial shape change, from regular round morphology to irregular shapes invading the 3D microenvironment (Figure 5a,b). Volumetric analysis of the 3D culture established that low‐volume progenitors, displaying a high average sphericity index, transform into large megakaryocytes, which significantly increase their volume while losing sphericity in favor of highly branched margins, reminiscent of proplatelet formation (Figure 5c). To better assess the dynamic of megakaryocyte metamorphosis, we performed the spatiotemporal volumetric imaging of samples in the last phase of differentiation. We observed that proplatelet formation starts with the elongation of plump pseudopodia. Next megakaryocytes increase their volume while losing sphericity, and platelets are released from their branching filaments (Figure 5d; Figure S3b; Videos S5–S7, Supporting Information). It is known that bone marrow megakaryocytes synthesize lipids and cholesterol that provide fluid membranes to elongate proplatelets.^[^ ^24^ ^]^ The 3D reconstructions of the cell membrane showed that proplatelets, enriched in cholesterol along their length, grow thin through the 3D space and actively release CD41a^+^CD42a^+^ platelet‐sized particles, as confirmed by in situ microscopy (Figure S3c, Supporting Information) and flow cytometry analysis of cells retrieved from the silk‐based construct after dissolution (Figure S3d, Supporting Information).

**Figure 6:**
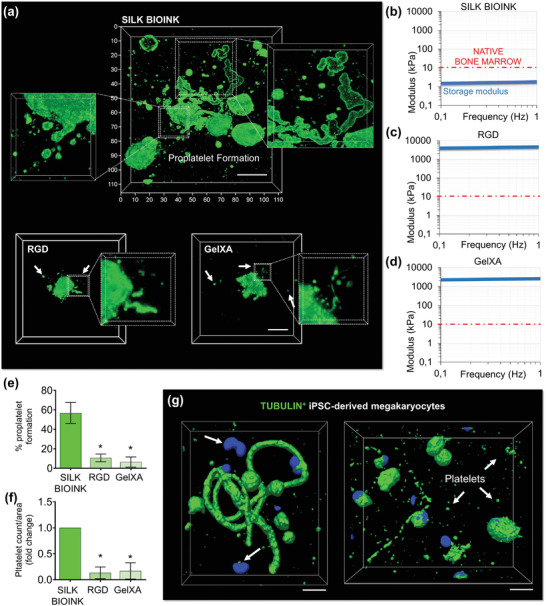
The unique softness of 3D bioprinted silk‐bioink is crucial to support thrombopoiesis. a), The silk bioink recreates a favorable environment for supporting increased proplatelet branching (top) compared to other conventional bioinks (bottom) (green = CD61; scale bar = 20 µm, representative of three independent experiments). b–d), Storage modulus of silk bioink compared to other conventional crosslinked bioinks b), silk bioink, c), RGD; d), GelXA). The red line identifies the maximal stiffness measured for the native bone marrow tissue. e), Percentage of proplatelet formation in the different tested conditions (n = 10; ^*^ *p*<0.001). f), Fold increase of platelet count/area in the different tested conditions (n = 10; ^*^ *p*<0.001). g), Human iPSC‐derived megakaryocytes (imMKCL) engineered to express fluorescent β1‐tubulin (green) show that proplatelet formation and platelet release are sustained by cytoskeleton remodeling (scale bar = 10 µm).

Over the course of live‐cell imaging studies, we observed that the release of platelets increases over time, and scattered CD41^+^ particles could be visualized throughout the silk bioink space (**Figure** **6**a; Videos S8 and S9, Supporting Information), further emphasizing the physiological relevance of our experimental system. Other commercial bioink formulations, used for comparison, were not able to support proplatelet formation and invasion into the 3D constructs (Figure 6a). Also, they contained megakaryocytes with fewer branches and a smaller number of released platelets nearby. Commercially available bioink have known limitations with respect to stiffness when compared to the native bone marrow, as they present a 100‐time higher storage modulus than silk bioink, whose softness, at 37 °C in standard culture conditions, is within the known ranges of the native hematopoietic niche (Figure 6b–d). The statistical assessment of the number of proplatelet forming‐megakaryocytes (Figure 6e) and released platelets (Figure 6f) collectively indicated that only the silk bioink could support physiological megakaryocyte function. The effectiveness of the proposed silk bioink formulation and bioprinting strategy was supported by the active cytoskeleton remodeling observed by the 3D reconstruction of β1‐tubulin microtubules which were shown to be distributed along the proplatelet shafts of human induced pluripotent stem cell (iPSC)‐derived megakaryocytes expressing the fluorescent protein (Figure 6g). Together, these data demonstrated that the silk bioink stands out as a soft natural bioink capable of supporting thrombopoiesis physiologically.

### 2.5 3D Modeling of Physiological and Pathological Megakaryopoiesis

Platelet disorders, affecting ≈ 3 million individuals worldwide, encompass a diverse range of rare diseases that can remain undiagnosed or misdiagnosed for years. Currently, Inherited Platelet Disorders involve multiple rare monogenic disorders resulting from mutations in >60 specific diagnostic‐grade genes.^[^ ^25^ ^]^ This genetic heterogeneity poses difficulties in establishing a standardized diagnostic approach for all cases, and the rarity of these diseases often limits expertise among healthcare professionals. Additionally, many regions lack access to nearby reference laboratories, further hindering the diagnostic process.

In this context, the analyses of platelet production and size play a crucial role in guiding the differential diagnosis.^[^ ^26^ ^]^ However, the rarity of these disorders makes it challenging to adequately train physicians, and conventional automatic counters may underestimate platelet volume and count.^[^ ^26^, ^27^ ^]^ Early and accurate diagnosis is crucial for prognosis determination, and identification of proper therapeutic strategies.

**Figure 7:**
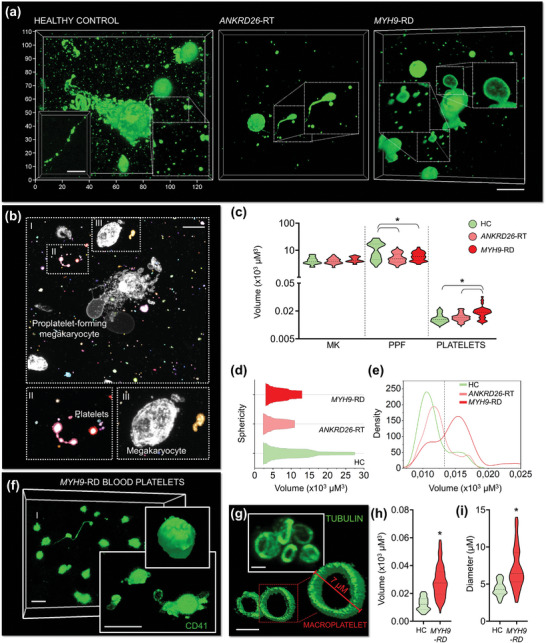
Silk bioink for disease modeling and diagnosis in Inherited Thrombocytopenia. a), Confocal microscopy analysis of proplatelet formation by 3D bioprinted megakaryocytes from healthy controls (HC) and patients affected by *ANKRD26*‐RT and *MYH9*‐RD (green = CD61; scale bar = 20 µm. Representative of n = 5 HC; n = 5 *ANKRD26*‐RT patients; n = 5 *MYH9*‐RD patients). b), Image segmentation analysis allows the discrete classification of mature round megakaryocytes (MK), proplatelet‐forming megakaryocytes (PPF), and released platelets (scale bar = 30 µm). c), Volumetric analysis of the different cell subpopulations (MK: n = 100; PPF: n = 100; platelets: n = 300; ^*^ *p*<0.001). d), Kite diagram of megakaryocyte volume and sphericity. **e**, Density plot of platelet volume. f), Representative confocal microscopy and rendering of platelets from the peripheral blood of patients affected by *MYH9*‐RD (green = CD41; scale bar = 8 µm. Representative of n = 3 *MYH9*‐RD patients). g), Representative confocal microscopy and rendering of β1‐tubulin staining of peripheral blood platelets of patients affected by *MYH9*‐RD (green = β1‐tubulin; scale bar = 5 µm. Representative of n = 3 *MYH9*‐RD patients). h), Volumetric analysis of 3D‐bioprinted platelets from the peripheral blood of patients affected by *MYH9*‐RD with respect to healthy controls (HC) (n = 150; ^*^ *p*<0.001). i), Analysis of peripheral blood platelet diameter of patients affected by *MYH9*‐RD and healthy controls (HC) 3D‐printed into the silk bioink (n = 100; ^*^ *p*<0.05).

To address these challenges, we researched to assess the effectiveness of the silk bioink in identifying specific phenotypic changes associated with Inherited Platelet Disorders. We applied our bioprinting approach to megakaryocytes derived from patients who had previously undergone diagnosis of *MYH9*‐Related Disease (RD) and *ANKRD26*‐Related Thrombocytopenia (RT), two forms of Inherited Thrombocytopenia characterized by moderate to severe low platelet count.^[^ ^28^, ^29^ ^]^ Our findings revealed significant morphological defects in the megakaryocytes from both patient groups, as well as a severe impairment in extending branched proplatelets throughout the silk bioink compared to healthy controls (**Figure** **7**a). The rate of cell differentiation and survival within the construct was comparable among all groups (Figure S4, Supporting Information).

Cell morphology serves as a valuable indicator of the underlying molecular mechanisms governing cellular functions, making it a useful tool for defining the pathological state of individual cells and informing medical decisions.^[^ ^30^ ^]^ Traditional approaches often result in information loss, but 3D culture, and imaging methods offer a comprehensive view of the entire cell surface. Utilizing automated image segmentation analysis, we calculated the volume of megakaryocytes and platelets produced within the 3D environment (Figure 7b). While the megakaryocyte volume was similar among patient samples, those with *MYH9*‐RD exhibited a lack of enlargement during proplatelet formation, consistent with their decreased branching capacity (Figure 7c). Also, the volume of platelets released by *MYH9*‐RD megakaryocytes was significantly higher than that observed in healthy controls and *ANKRD26*‐RT samples (Figure 7c). This aligns with the characteristic enlarged size of *MYH9*‐RD platelets.^[^ ^26^ ^]^

Based on this information, we established the proof‐of‐concept of a diagnostic approach for easy identification of pathological thrombopoiesis. Inherited Platelet Disorders could be classified based on the lowest volume‐to‐sphericity ratio (Figure 7d), with *MYH9*‐RD samples characterized by a higher density of released platelets with enlarged size (Figure 7e). Notably, the membrane (Figure 7f) and cytoskeleton staining (Figure 7g) of platelets obtained from peripheral blood samples of the same patients confirmed the effectiveness of the approach in distinguishing pathological samples with abnormally increased volume and diameter (Figure 7h,i).

### 2.6 Colorimetric Assay to Detect Platelet Formation in the Silk Bioink

**Figure 8:**
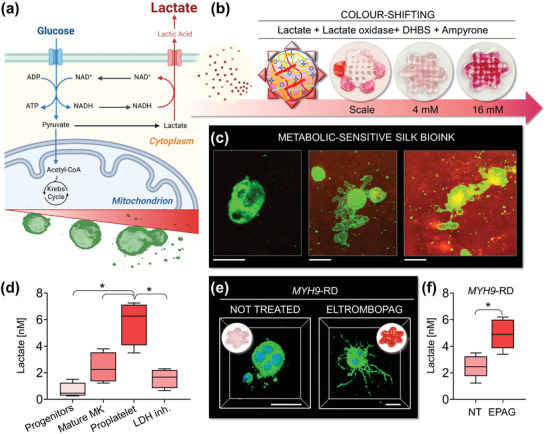
Silk bioink for easy‐readable detection of platelet production. a), Schematic of megakaryocyte metabolism. During differentiation and platelet formation, megakaryocytes undergo an increase of glycolytic activity and consequent release of lactate. Cartoon made using BioRender.com b), The silk bioink functionalized with horseradish peroxidase (HRP), lactate oxidase, and colorimetric sensors sense lactate concentrations. When lactate oxidase converts lactate into pyruvate and hydrogen peroxide, this latter is used as a cofactor by HRP that leads to a colorimetric shift of the silk bioink formulation in the presence of DHBS, and 4‐aminoantipyrine (4‐AAP). c), Imaging of silk bioink after the colorimetric reaction (green = CD61; scale bar = 20 µm). Colorimetric variations can be detected. The most intense color‐shift is detected at the stage of platelet production. d), A significantly increased release of lactate can be quantified during proplatelet formation by healthy megakaryocytes. A lactate dehydrogenase inhibitor (LDH‐inh.) has been used as control to assess the specificity of the colorimetric reaction (n = 5; ^*^ *p*<0.01). e), Silk bioink supports the 3D bioprinting of *MYH9*‐RD megakaryocytes. Treatment with the TPO‐RA Eltrombopag promotes increased proplatelet branching of *MYH9*‐RD megakaryocytes with respect to the untreated condition (green = CD42b; blue = nuclei; scale bar = 30 µm). f), Lactate levels are low in thrombocytopenic states (NT = not treated). Treatment with Eltrombopag increases the production of lactate (n = 5 *MYH9*‐RD patients; ^*^ *p*<0.05).

Recent studies have explored the use of silk‐based formulations to preserve the enzymatic activity of colorimetric sensors to create bio‐reactive fabrics.^[^ ^11^ ^]^ We have extended this potential to the silk bioink by incorporating enzymatic reactions directly into the formulation to track cell metabolism via colorimetric quantifications capable of providing a standardized, rapid, and non‐destructive measurement of cell function and platelet production into the 3D construct. Lactate level was chosen as a test indicator due to the intimate connection between megakaryocyte glucose metabolism and platelet generation (**Figure** **8**a).^[^ ^31^ ^]^ The glycolytic pathway and the Krebs cycle play crucial roles in supporting lineage specification, nuclear multiplication, and the accumulation of membranes necessary for successful megakaryocyte transformation into functional platelets, facilitating glycoprotein, nucleotide, and lipid synthesis.^[^ ^32^ ^]^ Traditionally, lactate variations are monitored in cell supernatants using lactate oxidase and horseradish peroxidase (HRP) enzymatic assays. However, these assays can be time‐consuming and lack standardization across different laboratories. Here, we incorporated HRP in the silk bioink formulation to evaluate the stability of the enzyme in its 3D‐printed format. The constructs were stored at 4, 37, or 70 °C for 15 days, and comparable chemiluminescence emission demonstrated the ability of silk‐based bioink to preserve overall enzymatic activity (Figure S5, Supporting Information). In contrast, other commercial bioink not containing silk when loaded with HRP showed nearly absent enzymatic activity after long‐term incubation, highlighting the breakthrough of the silk‐based approach (Figure S5, Supporting Information). To assess the sensitivity of the HRP^+^ silk bioink to lactate variations and create calibration curves, we exposed the constructs to increasing concentrations of lactate in the presence of lactate oxidase (Figure 8b). The silk‐based constructs exhibited uniform color changes. The intensity of color grading was increased proportionally to the concentration of lactate. When the HRP^+^ silk bioink containing differentiating megakaryocytes were incubated with lactate oxidase, we initiated an enzymatic reaction that led to colorimetric changes in the silk bioink. The extent of the signal increased according to the stage of megakaryocyte maturation. Confocal imaging combined with transmitted light imaging demonstrated localized activities of the sensing pattern in areas of robust platelet formation (Figure 8c). The most intense signal was observed throughout the silk bioink at the stage of proplatelet formation (Figure 8d). When samples were treated with a lactate dehydrogenase inhibitor, the amount of lactate decreased significantly, resulting in a reduction of the colorimetric signal, and confirming the specificity of the reaction.

To demonstrate its applicability in the clinic, we utilized the silk bioink to evaluate the response of *MYH9*‐RD megakaryocytes to Eltrombopag, a TPO‐receptor agonist (TPO‐RA) commonly used in clinical practice for the treatment of various forms of thrombocytopenia.^[^ ^33^ ^]^ High‐resolution imaging of silk bioink revealed increased production of highly branched proplatelets when *MYH9*‐RD megakaryocytes were exposed to the TPO‐RA (Figure 8e). Colorimetric analysis of the constructs showed a significant color‐shifting to intense signal after treatment with the TPO‐RA (Figure 8e), demonstrating that improved cell functionality is paralleled by increased lactate production when compared to the untreated counterpart (Figure 8f). In summary, these findings highlight the promising potential of silk bioink as an innovative diagnostic approach for platelet disorders. Our research underscores the effectiveness of integrating physiologically relevant 3D silk culture systems with high‐resolution imaging and metabolic sensing techniques, which could automate and standardize the analysis of human thrombopoiesis in an ex vivo setting. Expanding this approach to other blood lineages holds significant promise for advancing research in hematology, potentially accelerating the development of personalized strategies for diagnosis and treatment.

## 3 Conclusion

In summary, through the utilization of the silk‐based bioink formulation, we have developed a comprehensive approach for 3D bioprinting human blood progenitor cells in a standardized and automated fashion. Our methodology has facilitated the creation of 3D models characterized by highly reproducible mechanical properties, structural integrity, and uniform cell distribution. These advancements have enhanced megakaryocyte function, leading to improved platelet production. Existing bioink formulations are not capable of supporting hematopoiesis. Our silk bioink stands out as the softest natural formulation able to support physiological platelet production from iPSCs‐derived and human primary megakaryocytes. This approach has proven effective in identifying pathological phenotypes associated with platelet‐related disorders. *MYH9*‐RD and *ANKRD26*‐RT served as a proof‐of‐concept for validating our approach. Leveraging the transparency of silk bioink allowed us to conduct high‐resolution imaging and morphological classification of cell size and shape, differentiating between healthy and diseased proplatelet‐forming megakaryocytes and platelets. Notably, unlike *MYH9*‐RD, *ANKRD26*‐RT is not characterized by enlarged platelet size, and there are no known specific markers associated with this condition. Consequently, the assessment of platelet production capacity and size plays a crucial role in identifying the disease phenotype and holds broader applicability to other forms of thrombocytopenia.

The incorporation of biochemical reporters into the formulation have allowed us to demonstrate the correlation between cell metabolism and platelet production both in physiology and pathology. This provides a rapid and reproducible method for investigating terminal megakaryocyte differentiation and proplatelet formation. While colorimetric sensing offers intuitive binary responses, its combination with image analysis techniques has the potential to enhance the specificity and sensitivity of the diagnostic process and guide the selection of suitable treatments. Overall, the unique amalgamation of non‐thrombogenicity, mechanical properties, transparency, bioactivity, and ease of processing positions silk fibroin as an exceptional component of our bioink, supporting the study of platelet formation in a 3D environment.

Artificial Intelligence methods, including machine learning and deep learning algorithms, are expected to improve patient care by assisting the prediction and diagnosis of diseases, medical research, and clinical education and training, especially for those pathologies whose study is based on image analysis.^[^ ^34^ ^]^ The bone marrow niche remains challenging to access using current imaging techniques, preventing the direct observation of the dynamics of physiological and pathological hematopoiesis in vivo. The findings from our study offer promising opportunities for advancing the development of novel algorithms to enhance diagnostics in hematological diseases. Our aim is not to replace traditional diagnostic methods, but rather to identify key features associated with bone marrow disorders. This knowledge can contribute to a deeper understanding of the underlying pathogenic mechanisms, guide the prioritization of strategies for drug development, and pave the way for high‐throughput screening of new therapeutic options directly on patients’ blood progenitors.

## 4 Experimental Section

### Preparation of the Silk Fibroin Solution

Silk fibroin aqueous solution was obtained from *Bombyx mori* (CREA – AA), as previously described.^[^ ^35^ ^]^ Briefly, dewormed cocoons were boiled in Na_2_CO_3_. The fibers were rinsed in ultrapure water and dried overnight. The dried fibers were solubilized in LiBr. The solubilized silk solution was dialyzed against distilled water using a Slide‐A‐Lyzer cassette (Thermo Scientific) with a 3500 MW cutoff for three days. Before usage, the silk solution was centrifuged at maximum speed to remove large particulates.

### Silk Fibroin‐Based Bioink Formulation

The bioink was prepared with an 8% w/v silk fibroin in a physiologic salt solution containing glucose and HEPES. Type A gelatin (15% w/v) and alginate (1% w/v) were incorporated under gentle magnetic stirring. The pH was adjusted to 7.4. Before bioprinting, the bioink formulation was centrifuged at 300xg at room temperature.

### Rheology

The rheological characterization of silk bioink, silk fibroin solution, and gelatin/alginate bioink was performed using the Discovery HR‐2 rheometer with a Peltier plate (TA Instruments). Four different rheological measurements were performed. An aluminum 20 mm parallel plate configuration with a 500 µm gap was used for the first three methods, while for the last method, a serrated steel 8 mm plate−plate configuration with a 1000 µm gap. *Flow sweeps*: the samples were conditioned for 60 s before the sweeps to reach the equilibrium temperature. The flow sweep was performed at different temperatures monitoring the complex viscosity as a function of shear rate in the range of 0.001 – 500 1 s^−1^. The complex viscosity dependency on shear rate was fitted through a Carreau–Yasuda model:

(1) $$\eta^{*}=\eta_{\infty }+\left(\eta_{0}-\eta_{\infty }\right){\left(1+{\left(\lambda *\omega \right)}^{a}\right)}^{\frac{n-1}{a}}$$

where η_0_ and η_∞_ are the steady shear and the infinite complex viscosity, λ is the relaxation time, *a* is the transition control factor and *n* is a power law index. *Temperature sweeps*: samples were conditioned for 120 s before the measurements to reach an equilibrium temperature. The sweeps were performed between 40 and 15 °C decreasing temperature with a linear ramp (1 °C min^−1^) up and keeping the strain (0.1%) and frequency (1 Hz) constant. *Thixotropic test*: the samples were conditioned for 120 s at 37 °C before the test. The test was divided into different complete‐time segments, each taking 160 s with a measurement time of 1 s per point. The shear rate was initially set at 0.01 1 s^−1^ and then ramped up to 100 1 s^−1^ to mimic bioink deformation under the pressure during printing and finally was brought back to 0.01 1 s^−1^ to evaluate the recovery after the deformation. *Oscillation frequency sweeps*: the tests were performed on 3D constructs after the crosslinking step at 1% strain. Samples were kept at 37 °C.

### Bioprinting Parameters

The silk bioink formulation was loaded into a sterile cartridge fitted with 18G, 20G, or 22G nozzles and kept at 37 °C in a BIO‐X extrusion bioprinter (Cellink). The thermo‐regulated printhead was equipped with an insulator that could house a nozzle/needle. This allowed precise control of the temperature over the needle to ensure temperature uniformity down to the print surface while keeping cells at their physiological conditions. The distance between the printing nozzle and the surface of the culture plate was set as 2/3 of the needle diameter. The print‐bed was set at 15–20 °C to allow fast solidification of the silk bioink after deposition on the culture plate. The printing process was performed with different cylindrical steel needles (18, 20, 22 G) at different pressures (8, 12, 16, 20 kPa). The combination of these parameters was tested at 6, 8, 10, and 12 mm s^−1^ printing speed. Under light microscopy, the printed shapes were transparent. An image of the deposited filaments at the different tested printing nozzle, pressure, and speed was captured with an Olympus microscope IX53 (Olympus) equipped with a 4x objective, and their widths were measured using Olympus image processing software.

### Elaboration and Bioprinting of the 3D Construct

Software Fusion 360 (AutoDesk) was designed with the desired pattern. The construct consisted of a layered hexagon intersected with 6 other smaller hexagons at its borders to shape a “*flower*”‐like model. The model can be tailored to have different dimensions, and then exported in Standard Triangulation Language (STL) format. The bioprinter integrates a slicer that allows to automatically converting the STL files to the G‐code for the printing process. The printing process of the bone marrow model was set at 12–16 kPa, 8–10 mm s ^−1^ speed with a 20 G needle. Constructs of different dimensions (1×1, 2×2, 3×3, 4×4 cm) were printed with a 25% grid infill. The presence of the grids has been designed to allow homogenous diffusion of medium and cytokines inside the scaffolds during cell culture, and the diffusion of antibodies and staining reagents during the analysis of the fixed samples. The height of each subsequent layer was set at 80% of the inner diameter of the needle to ensure proper adhesion between layers. After bioprinting, the scaffold was crosslinked for a minimum of 10 min in a physiological salt solution containing CaCl_2_. Then, the 3D construct can be immersed in a cell culture medium and kept at 37 °C and 5% CO_2_.

### Primary Cell Culture

Human umbilical cord blood was collected following physiologic pregnancies and deliveries upon informed consent of the parents. Human peripheral blood samples were obtained from healthy controls and thrombocytopenic patients after informed consent. Patients had previously undergone diagnosis through whole‐exome sequencing, as previously described.^[^ ^29^ ^]^ All samples were processed following the ethical committee of the I.R.C.C.S. Policlinico San Matteo Foundation (2017‐0003984) and the principles of the Helsinki Declaration. CD34^+^ or CD45^+^ cells were separated by an immunomagnetic bead selection kit (Miltenyi Biotec), as previously described.^[^ ^16^, ^36^ ^]^ Samples were differentiated in Stem Span medium (StemCell Technologies) supplemented with 1% penicillin‐streptomycin (P/S), 1% L‐glutamine, 10 ng/mL Thrombopoietin (TPO) and interleukin (IL)−11 (Peprotech).

The culture of immortalized megakaryocyte progenitor cell line (imMKCL) from induced pluripotent stem cell‐derived hematopoietic progenitors was performed as previously described.^[^ ^37^ ^]^ Dox‐ON proliferation culture: imMKCLs were cultured in the presence of 5 µg/mL doxycycline to induce proliferation due to the expression of c‐MYC, BMI1, and BCL‐XL. imMKCLs were cultured in a humidified incubator at 37 °C and 5% CO_2_ in basal medium supplemented with 15% fetal bovine serum, 50 ng mL^−1^ stem cell factor (SCF), 50 ng mL^−1^ TPO. The basal medium consisted of IMDM supplemented with L‐glutamine, Insulin‐transferrin‐selenium, 50 µg mL^−1^ Ascorbic acid, and 450 µm 1‐thioglycerol. Dox‐on culture was performed in classic liquid medium. Dox‐OFF differentiation culture: the culture for imMKCL maturation to platelets was done for 5 days in basal medium supplemented with 50 ng mL^−1^ SCF, 50 ng mL^−1^ TPO, 15 µm KP‐457, 0.75 µm SR‐1, 10 µm Y27632 and 15% FBS. The dox‐off culture was performed into the silk bioink.

### Cell Bioprinting

Megakaryocyte progenitors, imMKCLs, or platelets were mixed with the silk bioink heated to 37 °C before usage. The silk bioink formulation was printed at 12 kPa, 8‐10 mm s ^−1^  speed with a 20 G needle to obtain a “*flower*” of 3–5 layers, 25% infill, and 3×3 cm dimension. After printing all the samples were crosslinked into a physiological salt solution containing CaCl_2_. The bioprinting efficiency was verified by comparing the composition to the formulation comprising only 8% w/v silk fibroin and 15% w/v type A gelatin (NO alginate), or 8% w/v silk fibroin and 1% w/v sodium alginate (NO silk). 3D bioprinted constructs were cultured in the respective medium, as described above. For silk bioink dissolution, samples were incubated into a formulation comprising sodium citrate, collagenase, and alginate lyase dissolved in physiologic salt solution containing glucose. The melting temperature was set at 37 °C for 10–20 min. In some experiments, cells were mixed into GelXA or CELLINK RGD bioink (Cellink) and 3D bioprinted according to manufacturer instructions.

### Assessment of Swelling

The analysis had been performed as previously described.^[^ ^38^ ^]^ 3D printed constructs underwent a process of patting dry after crosslinking and were initially weighed as W_i_. Subsequently, they were immersed in the culture medium and maintained at 37 °C with 5% CO_2_ for 7 days. At regular intervals of 24 h, samples were carefully retrieved, patted dry once again, and weighed as W_f_. The swelling ratio (S) was then calculated using the equation S = W_f_/W_i_ ×100. For constructs lacking alginate, rapid dissolution into the culture medium occurred, preventing accurate weight measurement.

### Imaging

The 3D‐printed silk bioink was housed in a customized device manufactured using 3D Stereolithography (LSA) printing technology (FormLab). The model was created using Fusion 360 (AutoDesk), and PreForm software was used for slicing (FormLab). The printing was done using a long‐term non‐toxic biocompatible resin (FormLab). A glass window at the bottom ensured possibilities for live‐cell imaging and high‐resolution microscopy. For immunofluorescence imaging samples were fixed in 4% formaldehyde for 20 min, at room temperature. Samples were probed with anti‐CD34 (1:100), anti‐CD61 (1:100), anti‐CD41 (1:100) (Beckman Coulter), CD42b (1:100) (Invitrogen), or anti‐β1‐tubulin (1:200) (Abcam), overnight at 4 °C. Alexa Fluor secondary antibody (1:500) (Invitrogen) have been incubated for 2 h at room temperature. Nuclei were stained with Hoechst 33 258 (Sigma–Aldrich). Samples were imaged by an SP8 confocal laser scanning microscope (Leica). For live imaging, samples were stained with FITC conjugated anti‐CD61 or anti‐CD41 antibodies, or Cytopainter Cell Plasma Membrane Staining Kit and NBD Cholesterol Staining Dye Kit (Abcam). Nuclei were stained with BioTracker NIR694 Nuclear Dye (Sigma‐Aldrich). Isotype controls were used as a negative control to exclude non‐specific background signals. The acquisition parameters were set on the negative controls.

### 3D Segmentation

Random forest classifiers for both platelets and megakaryocytes were trained in Napari using APOC. These classifiers were used in an initial semantic segmentation step, which was followed by connected component labeling using sci‐kit‐image. For platelet segmentation, objects smaller than 50 voxels and larger than 80 000 voxels were filtered out, and the remaining objects were counted by determining the largest‐area slice in the 3D volume and measuring the length of the long axis of this 2D slice. For megakaryocyte segmentation, objects smaller than 100 000 voxels were removed. Object Volume was determined by multiplying the number of voxels by the voxel size. The surface area was determined by mapping a surface using the marching cubes algorithm and then calculating the surface area of the resulting mesh using sci‐kit‐image. Alternatively, 3D reconstruction and image processing were performed using Leica LasX (Leica) and Arivis Vision 4D (Zeiss). Sphericity and volumes were measured by Arivis Vision 4D (Zeiss). The analyses of pathological samples have been performed blinded to patients’ diagnoses.

### Responsive Bioink Preparation

Responsive silk bioink was realized by mixing the formulation with 340 U mL^−1^ HRP. The bioink was mixed with 1×10^6^ cells mL^−1^ and bioprinted following the settings described above. During different days of culture, the whole construct or part of it was immersed in a solution containing lactate oxidase (LOX), DHBS, and 4‐aminoantipyrine (4‐AAP). In the presence of lactate, the resulting reaction produces pyruvate and hydrogen peroxide. The latter reacts with HRP causing a change in the color of the printed scaffold, proportional to the quantity of lactate present in the scaffold. Incubation with the lactate dehydrogenase A inhibitor FX11 (Merck) have been used as control. A standard reference curve has been estimated by mixing a known concentration of lactate with the silk bioink. In parallel, the reaction has been performed in liquid medium in a conventional well plate to obtain a standard calibration curve, spanning from 0 to 20 mM lactate. The colorimetric output has been measured on a microplate reader (POLARstar Omega, BMG LABTECH). Values proved to be perfectly comparable.

Immunofluorescent samples were visualized into colored bioink by SP8 confocal laser scanning microscope (Leica). Transmitted light images were recorded concurrently with the 633 nm red, 561 nm green, and 488 nm blue lasers, with these images pseudocolored and combined into an overlay image that matched the color camera image.^[^ ^39^ ^]^

### Flow Cytometry

Megakaryocytes and platelets were retrieved from the silk bioink by using the solution for dissolving the 3D construct described above. Flow cytometry settings were established, as previously described.^[^ ^14^ ^]^ Megakaryocyte ploidy, expression of surface markers, and viability were analyzed, as previously described.^[^ ^40^ ^]^ All antibodies were from BioLegend. Platelets were analyzed using the same forward and side scatter pattern as human peripheral blood and identified as CD41a^+^CD42a^+^ (BioLegend) events. Isotype controls were used as a negative control to exclude non‐specific background signals. All samples were acquired with a BD FACS Lyric (Becton Dickinson) flow cytometer. Off‐line data analysis was performed using the Kaluza software package (Beckman Coulter).

### RT‐PCR

Total RNA was extracted using the Mammalian GeneElute total RNA kit (Sigma‐Aldrich). Retrotrascription (RT) was performed using the iScriptTM cDNA Synthesis Kit according to the manufacturer's instructions (BioRad). For quantitative Real‐Time PCR, RT samples were diluted up to three times with ddH_2_O, and the resulting cDNA was amplified in triplicate with 200 nm of primers and SsoFast Evagreen Supermix (BioRad). The amplification was performed in a CFX Real‐time system (BioRad) as follows 95 °C for 5′, 35 cycles at 95 °C for 10′’, 60 °C for 15′’, and 72 °C for 20′’. Predesigned KiCqStart primers were purchased from Sigma‐Aldrich. The BioRad CFX Manager software 3.0 was used for the normalization of the samples (BioRad). GAPDH gene expression was used for comparative quantitative analysis

### Statistics

Values are expressed as mean plus or minus standard deviation or median and range. The Student *t*‐test or 1‐way analysis of variance (ANOVA) followed by Bonferroni posttest were used to analyze experiments. A p value of at least 0.05 was considered statistically significant. All experiments were independently repeated at least 3 times. The number of repetitions is specified in each figure legend. GraphPad Prism and OriginPro software have been used for graph representation and statistical analysis.

### Ethics Approval Statement and Patient Consent Statement

Blood samples were obtained after informed consent and processed following the ethical committee of the Istituto di Ricovero e Cura a Carattere Scientifico (I.R.C.C.S.) Policlinico San Matteo Foundation and the principles of the Helsinki Declaration

## Conflict of Interest

C.A.D.B., V.K., P‐A.L., I.N.R., and A.B. have submitted a patent application associated with this work. The other authors declare no conflict of interest.

## Author Contributions

C.A.D.B. designed research studies, conducted experiments, acquired and analyzed data, and wrote the manuscript; M.L. and V.K. conducted experiments, acquired and analyzed data, and wrote the manuscript; P‐A.L. conducted experiments; G.D.R. analyzed data and edited the manuscript; D.E.D.N. and F.J. provided technical support for image analysis and edited the manuscript; K.E. provided iPSC samples and edited the manuscript; C.D.F., C.P., and A.P. provided blood samples and edited the manuscript; I.N.R. provided technical support, analyzed data, and edited the manuscript; A.B. conceived the idea, supervised the project, designed research studies, analyzed data, and wrote the manuscript

## Supporting information

Supporting Information

Supplemental Video 1

Supplemental Video 2

Supplemental Video 3

Supplemental Video 4

Supplemental Video 5

Supplemental Video 6

Supplemental Video 7

Supplemental Video 8

Supplemental Video 9

## Data Availability

The data that support the findings of this study are available from the corresponding author upon reasonable request.
