# Supplementary material for: Bioprinting Soft 3D Models of Hematopoiesis using Natural Silk Fibroin‐Based Bioink Efficiently Supports Platelet Differentiation
Source: Adv Sci (Weinh). 2024 Mar 21;11(18):2308276. doi: 10.1002/advs.202308276 (PMC11095152; doi:10.1002/advs.202308276)
Supplement: Supplementary file 1 — Supporting Information [file ADVS-11-2308276-s010.pdf]

ADVANCED SCIENCE
Open Access

Supporting Information

for *Adv. Sci.*, DOI 10.1002/advs.202308276

Bioprinting Soft 3D Models of Hematopoiesis using Natural Silk Fibroin-Based Bioink Efficiently Supports Platelet Differentiation

*Christian Andrea Di Buduo, Marco Lunghi, Volodymyr Kuzmenko, Pierre-Alexandre Laurent, Giulia Della Rosa, Claudia Del Fante, Damian Edward Dalle Nogare, Florian Jug, Cesare Perotti, Koji Eto, Alessandro Pecci, Itedale Namro Redwan and Alessandra Balduini**

Supporting Information

Bioprinting soft 3D models of hematopoiesis using natural silk fibroin-based bioink efficiently supports platelet differentiation.

*Christian Andrea Di Buduo[a], Marco Lunghi[a], Volodymyr Kuzmenko[b], Pierre-Alexandre Laurent[a], Giulia Della Rosa[a], Claudia Del Fante[c], Damian Edward Dalle Nogare[d], Florian Jug[d], Cesare Perotti[c], Koji Eto[e,f], Alessandro Pecci[g], Itedale Namro Redwan[b], Alessandra Balduini[a,h]**

[a] C.A. Di Buduo, M. Lunghi, P-A. Laurent, G. Della Rosa, Prof. A. Balduini*
Department of Molecular Medicine, University of Pavia, 27100, Pavia, Italy
* E-mail: alessandra.balduini@unipv.it

[b] V. Kuzmenko, I. Namro Redwan
CELLINK Bioprinting AB, 412 76, Gothenburg, Sweden

[c] C. Del Fante, C. Perotti
Immunohaematology and Transfusion Service, Fondazione I.R.C.C.S. Policlinico S. Matteo Foundation, 27100, Pavia, Italy

[d] D.E. Dalle Nogare, F. Jug
Human Technopole, 20157, Milan, Italy

[e] Prof. K. Eto
Department of Clinical Application, Center for iPS Cell Research and Application (CiRA), Kyoto University, 606-8507, Kyoto, Japan

[f] Prof. K. Eto
Department of Regenerative Medicine, Graduate School of Medicine, Chiba University, 260-8670, Chiba, Japan

[g] Prof. A. Pecci
Department of Internal Medicine, I.R.C.C.S. Policlinico S. Matteo Foundation and University of Pavia, 27100, Pavia, Italy

[h] Prof. A. Balduini
Department of Biomedical Engineering, Tufts University, 02155, Medford, MA, USA
* E-mail: alessandra.balduini@tufts.edu

Supplemental Figures and Figure Legends

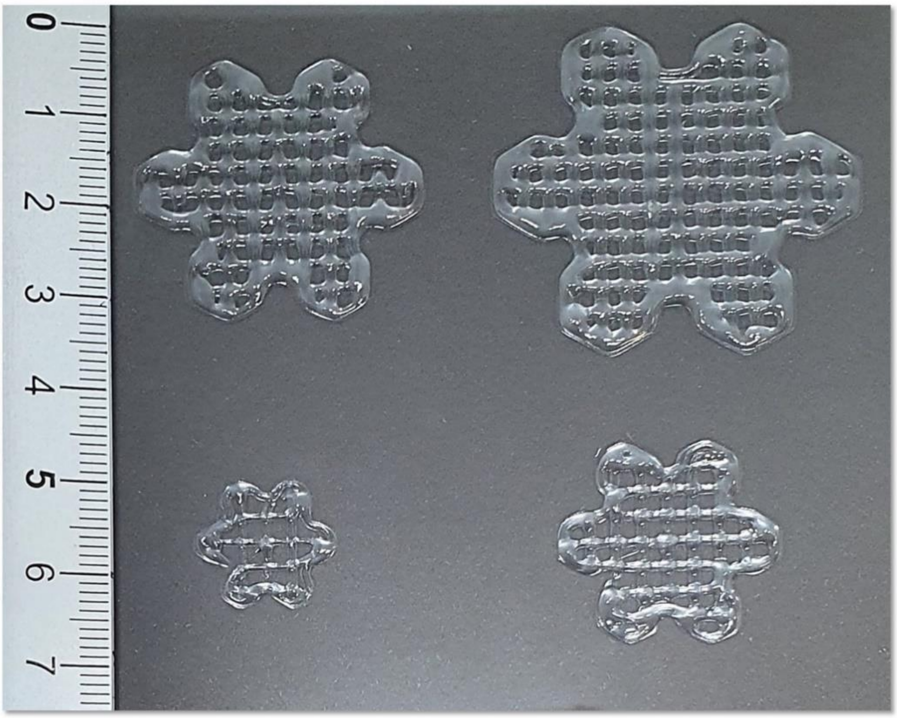

Figure S1. **Silk-‘*flower*’ printability**. Silk bioink can be used to obtain a wide range of different dimensions while maintaining high shape fidelity.

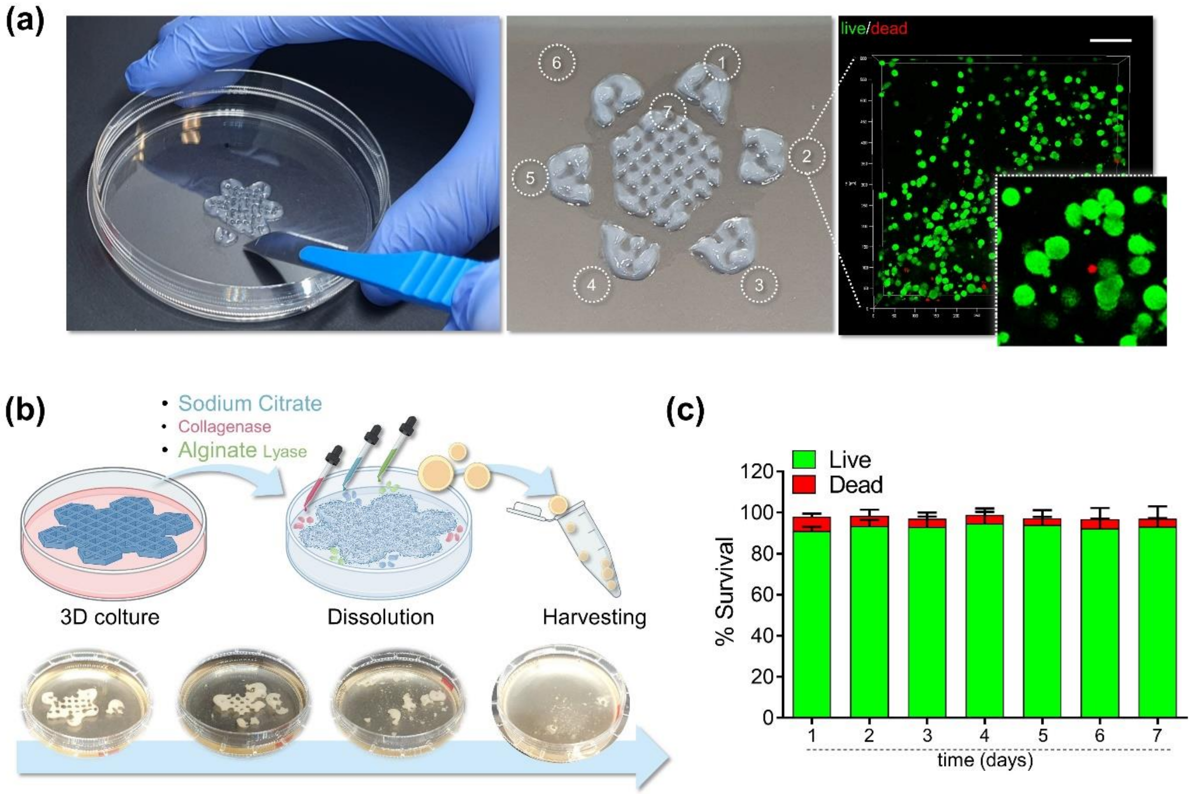

Figure S2. Viability of HSPCs into the 3D bioprinted construct. a), Primary human HSPCs are 3D bioprinted into the construct. The ‘*flower*’-like design allows to cut the petals over the course of the culture to perform confocal microscopy analysis without sacrifice the entire structure (green = live; red = dead; scale bar = 100 µm). b), To allow the release and harvesting of cells from the 3D culture, the 3D bioprinted construct was dissolved in a specific buffer containing sodium citrate, alginate lyase and collagenase. c), Statistical analysis of live/dead staining from retrieved HSPCs (n = 3; p = NS).

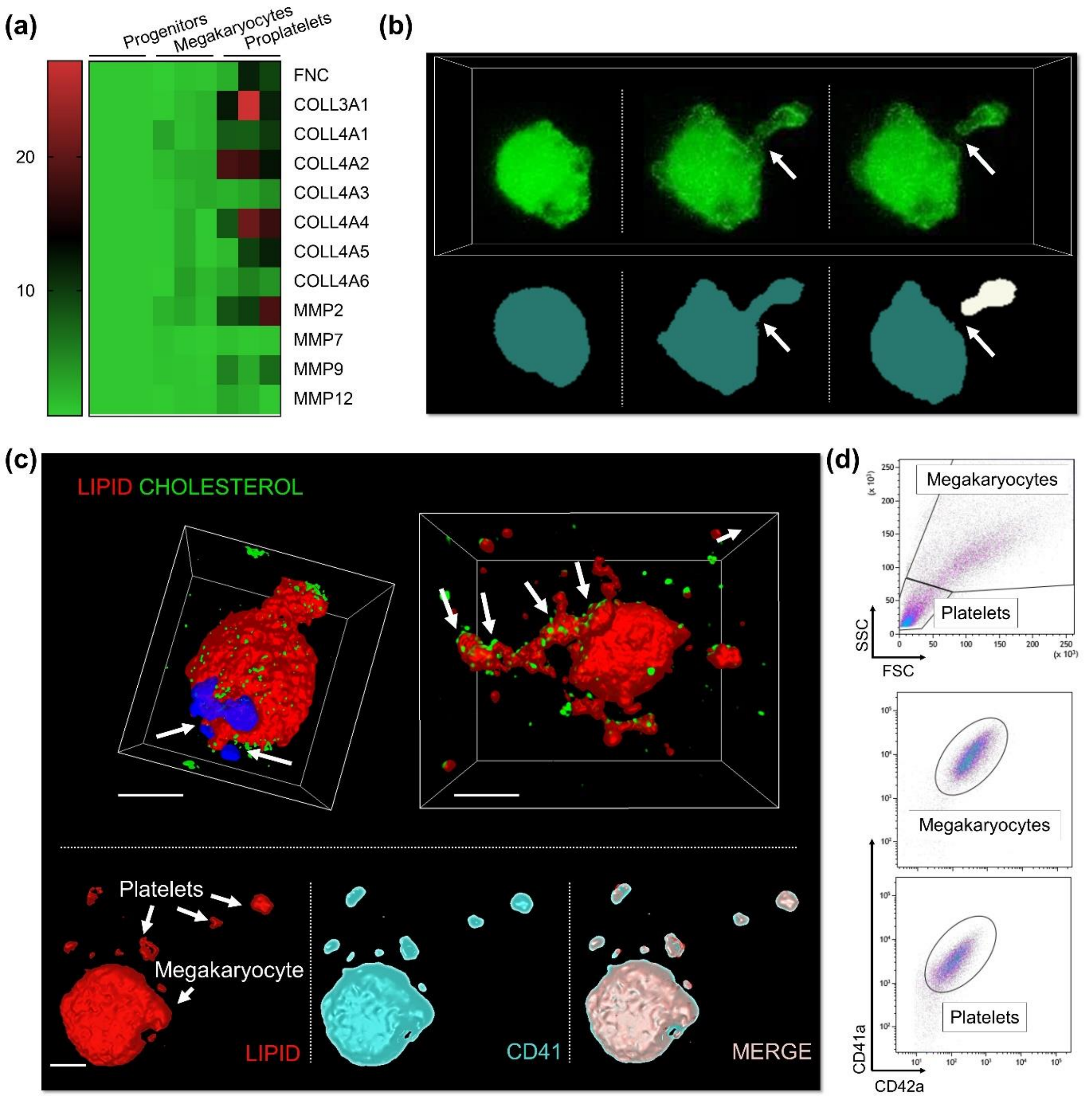

Figure S3. **Megakaryocyte characterization into the 3D bioprinted construct**. a), Real Time-PCR analysis for the expression of genes for extracellular matrix components and matrix remodeling enzymes (n = 3). b), Spatiotemporal volumetric imaging of a megakaryocyte extruding proplatelet and releasing platelet into the 3D construct. c), Staining of megakaryocyte membranes with probes for live imaging of cholesterol (green) and lipids (red) (blue = nuclei; 10 µm). The proplatelet shaft is enriched in cholesterol. Lipid (red) and CD41 (cyan) co-localize at the surface of both megakaryocytes and platelets (scale bar = 5 µm). d), Representative flow cytometry analysis of platelets and megakaryocytes retrieved from the dissolved construct.

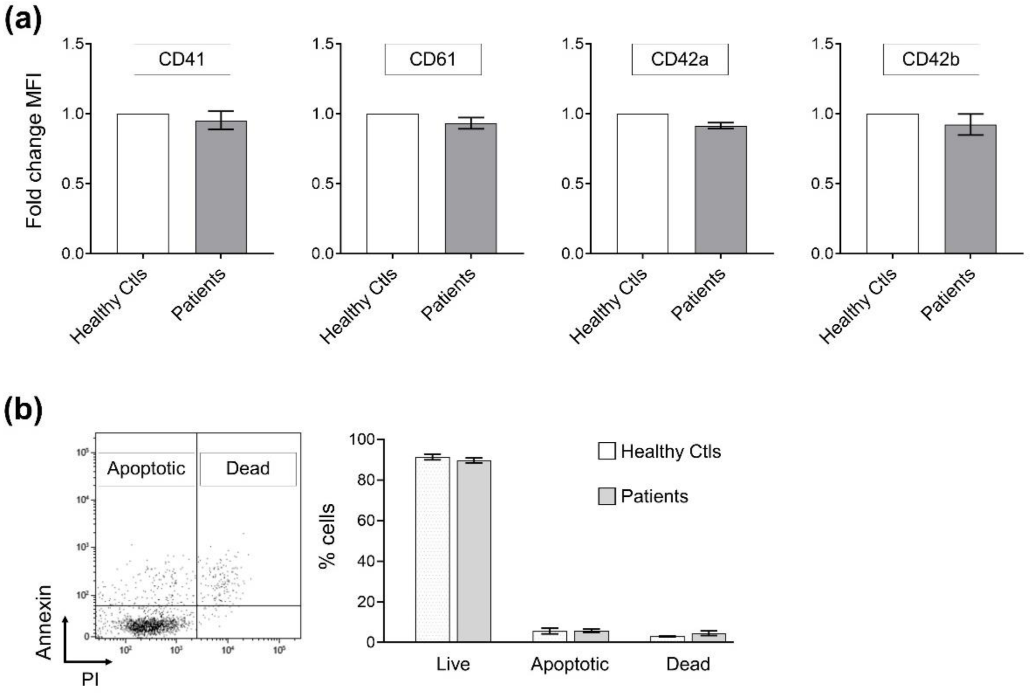

Figure S4. **Assessment of patient megakaryocyte differentiation**. a), Flow cytometry analysis of patient-derived megakaryocytes retrieved from the silk bioink shows no differences in expression of the lineage-specific surface differentiation markers when compared to healthy controls (n = 5 Healthy Controls; n = 10 patients; p = NS). b), Annexin/Propidium (PI) assay shows comparable numbers of viable cells between healthy controls and patients.

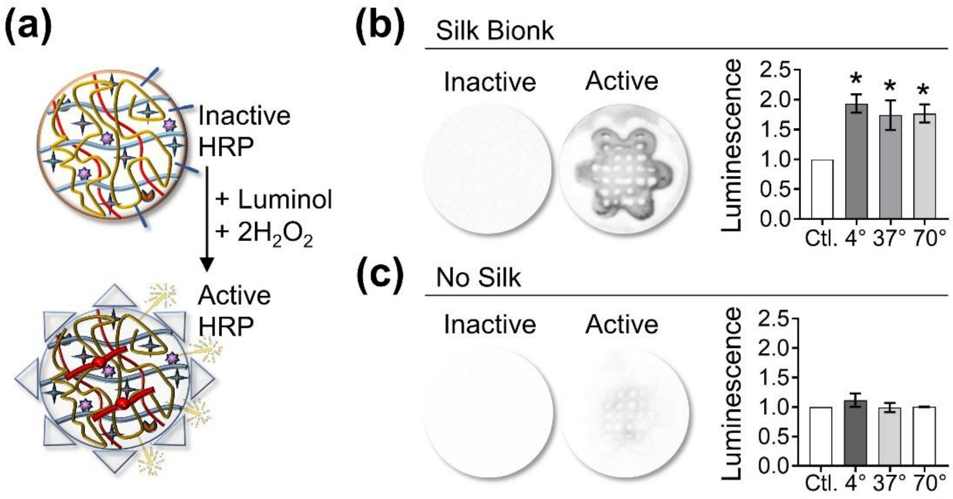

Figure S5. **Assessment of silk bioink functionalization with enzymes.** a), To evaluate the ability of silk bioink to preserve the activity of enzymes, horseradish peroxidase (HRP) was embedded into the silk bioink formulation or other commercial bioink as a control. Luminol and hydrogen peroxide were dropped onto the system to activate a chemiluminescent reaction. b), Silk bioink was able to preserve the enzymatic activity of HRP when embedded into 3D constructs kept at different temperatures. c), Other commercial bioink could not maintain HRP activity ($n = 3$; $p<0.01$).
